# Mental Dynamics, or Groundwork of a Professional Education. The Hunterian Oration, before the Royal College of Surgeons of England, 15th February, 1847

**Published:** 1847-07

**Authors:** 


### ART. XIV.

*Mental Dynamics, or Groundwork of a Professional Education. The Hunterian Oration, before the Royal College of Surgeons of England, 15th February,* 1847. By JOSEPH HENRY GREEN, F.R.S.—*London,* 1847. 8vo, pp. 64.

HERE is a disciple of a philosopher in the antique spirit, uttering those truths which he has imbibed from the conversations of the sage. If there was a modern man who realized "the old masters of the Academy or Lyceum," who in these times when all instruction is conveyed in books or in public speaking, was yet more eloquent, and full and instructive in conversation, and to a band of affectionate and trusting disciples imparted lessons which they received as oracles, it was Coleridge, the guiding spirit of Mr. Green's mind. "Throughout a long-drawn summer's day," says one of these disciples, "would this man talk to you in low, equable, but clear and musical tones, concerning things human and divine; marshalling all history, harmonizing all experiment, probing the depths of your consciousness, and revealing visions of glory, and of terror to the imagination; but pouring withal such floods of light upon the mind, that you might for a season, like Paul, become blind in the very act of conversion. With a calm mastery over your soul, leading you onward and onward for ever through a thousand windings, yet with no pause, to some magnificent point in which, as in a focus, all the particoloured rays of his discourse should converge in light."* To have been the intimate

* Table Talk of S. T. Coleridge, by H. M. Coleridge. Preface, xvi.

friend, and the favoured disciple of such a man during the latter part of his life, and to have been left the literary executor of his unfinished and most valued work, was the high honour of Mr. Green; and, taking this public occasion in which he was called on, under somewhat peculiar circumstances, to deliver an oration to the honour of Hunter, he has paid the worthiest tribute to his own teacher in philosophy by enforcing those truths which it was the life's object of that great man to proclaim. There was something peculiarly appropriate in joining the names of Coleridge and of Hunter. For, as all readers of 'The Friend' remember, Coleridge at an early period of this century comprehended Hunter's idea, and the purport of his museum in unfolding it, and proving it, and he correctly estimated the effect of these physiological views on practical Medicine and Surgery, and on the progress of comparative Anatomy. (See 'The Friend,' vol. iii, pages 181 and 215, 2d edit.) That he should have expressed these conclusions, and many times repeated his discriminative and high eulogies on Hunter in a work, the object of which was to exhibit the true method of all investigation, scientific and philosophical, is quite a sufficient reason for bringing the names of Coleridge and Hunter together, and for introducing, even in an Hunterian Oration, the name of a poet and a philosopher as an authority for the principles on which a professional education should be conducted.

"Hunter's peculiar merit," says Mr. Green, "was to have first fixed the principles of physiology, by banishing hypotheses, fictions, and arbitrary assumptions, and by considering life as a law;—implying that it is a power anterior, in the order of thought, to organization, which yet it animates, sustains, and repairs—a power originative and constructive of organization, in which it continues to manifest itself in all the forms and functions of living being." (pp. 6-7.)

We are as ready and willing as Mr. Green to testify at all times to the surpassing merits of Mr. Hunter; but we must demur to the adequacy of the illustration selected: the "peculiar merit" here held up for our homage, we believe to be a positive error. But the orator's purpose was not so much to consider Hunter's merit as a physiologist or a surgeon, but as a pattern for the formation of a scientific character. And here the difficulty presents itself, that as the characteristic of Hunter's mind was genius, and if genius is looked upon as a special gift of Providence imparting to its possessor somewhat of the creative power of the Divine giver, it cannot then be acquired by mental culture, and is no example for our imitation. But instead of thus viewing genius, our professor regards it as the healthy balance and harmonious development of all the mental faculties, moral and intellectual, brought into full action by the persevering energy of a constant will. It is this energy of character, with genial power, which stamps an indelible unity on the works and acts of Luther, of Dante, of Milton. But not a mere lawless will, but a will under the influence of the conscience, a moral will, for the powers of the mind cannot be harmoniously developed unless the will is subjected to this imperative law. Such a man feels that his aim in life, the very purpose of his existence here, is to cultivate by strenuous and indefatigable exertion those powers, moral and intellectual, which are the birthright of our humanity: that this is the goal to which he must strive to approach—this is the ideal man. To attain or rather to approximate to this point, is the object of "a liberal education," and the purport of this Oration is "to define the intellectual discipline which will best prepare the mind for the scientific cultivation of a profession, and aid the individual in forming his character in the light of a final aim." The golden rule of all systematic education, according to Mr. Coleridge and his pupil, is, that all living knowledge proceeds from within the man; that it may be trained, fed, and excited from without through the senses by instruction, but not infused or impressed by such means; that his real mental progress will depend upon his own exertion of the inward powers which have been thus roused and trained. Hence the necessity of disciplining the mind to habits of reflection, and to those branches termed Abstraction and Generalization. How early these faculties come into play is seen in the child, who, in his perception of things that are alike, abstracts what is like in any object from what is different, and by associating these like objects together, he generalizes.

Words are the earliest products of these powers, and the commencement of education. Hence the importance of grammar, which teaches the first lessons in thinking of the meanings of words, and initiates in the art of arranging words and sentences perspicuously, so that "it would be difficult to attach too much importance to a knowledge of words, their definite import, and right use, as grounded in grammar and evinced by a correct style." Such discipline leads to the acquirement of many kinds of knowledge which assist that great purpose ever to be kept in view, the harmonious development of the whole mind.

"Thus, as admirably suited to engage and exercise all the faculties, which may be supposed to be predominant, active, or capable of further evolution at an early period of mental training—we may mention natural history, considered here without especial reference to its scientific character—a study, which offers food for every digestion, knowledge for every capacity, and which tends more perhaps than any other to people, to enlarge, and to tranquillize the mind." (p. 18.)

Here curiosity is ever excited, attention riveted, and memory bribed by perpetual novelty, variety, and beauty. The comparing power is ever kept alive by an endless succession of similitudes and contrasts; and the reasoning power is finally evolved in order to trace and to explain the adaptation of means to proximate ends in instincts, which, when enlightened by reason, become in man the human understanding.

"Thus, as the student watches the ascension of nature into mind, he shall learn that, up the whole ascent, nature is a prophetic-hymn, heralding the advent of man, and proclaiming the wisdom and goodness of the Creator." (p. 19.)

As natural history is nearly conjoined with the history of man by a knowledge of the earth as his abode, a knowledge of geology prepares the way for that of the history of man.

"His primæval state, the separation of races, the gathering and swarming of hordes, the settlement of nations, and the formation of political confederacies and states—history, exhibited as the great scheme of Providence, which has been, and ever is, operative in the moral education of man, considered as the mind and soul of the planet." (p. 21.)

Looking back at the races of men we see one momentous difference—

"A certain number of nations, or people of the earth, retain the conditions of progress, while the remaining parts of the species bear the decisive stamp of their inferiority, their improgressiveness; communities, whose to-day is an everlasting yesterday, or varied only by the accidents of the day, more or fewer calamities, less or greater wretchedness. The proximate cause of this awful fact is evidently this.—The nations that are historic and progressive, contain in themselves the means and instrument by which their progress may be effectuated, namely, literature;—literature as connecting one nation with others diversely though equally cultivated, and as connecting the present with the past and with the future. They possess that which gives a continuity to time, and emancipates both individuals and communities from the hard necessity of existing as successive fragments,—like a glow-worm in the scanty circle of its own light, all before and all behind and, save in the narrowest vicinity, all around—in impenetrable darkness. But literature, thus regarded in its high vocation of representing the total mind of a nation or people, no nation can have without a living intercommunion with the master minds of all ages and countries. Need I say that this communion, this intelligible converse, whether we speak of the nation collectively, or of the individuals of which it is composed, can only be the result of a familiarity with the language, which is the medium of communication, and with the words, which are the living educts wherein the productive thoughts are embodied? Any additional language, of which a man is master, is as it were a new limb without its deformity. And I scarcely need dilate on the importance of that which enables a man to collect into his own individuality the discoveries, the mental wealth, the ennobling affections, and the models of the wise and great of countries and states under the most auspicious circumstances; or on the moral utility of an instrument, which may enable him to confer a portion of his knowledge and power on the inhabitants of less fortunate countries." (pp. 22-3.)

The study, therefore, of languages of these "literæ humaniores," is indispensable to explain the process by which the present state of intellectual cultere has been attained, and "to arm the mind for future enterprise and further conquests." "How shall we correctly interpret the oracles of God without a critical study of the languages in which they were recorded, written as they were for our instruction in that which concerns every man who is not wanting to himself?" How shall we learn political science unless we read the historians of antiquity? "What reliable knowledge of philosophy or of speculative science, without invoking the genius of Plato, and Aristotle? What hope of a cultivated taste without a familiarity with the excellencies of the models of ancient Greece, with a view to their fruitful comparison with the master-works of modern genius?" Such knowledge cannot be acquired by all in equal proportion and in early youth, but the sketch may serve as a comprehensive survey to an education which has claims to the term "liberal."

The orator assumes that such knowledges obtained in public schools may be considerable. Their use is to educate the intellect for the real business of life, so that a mind thus furnished and where powers have been thus strengthened, may be used as an instrument for the attainment of that practical knowledge and skill which the future sphere or profession of the individual may require. And as grammatical studies exercise the powers of analysis and synthesis, and as all sound judgment depends on these mental functions, the connexion of languages with a practical education (the *cui bono* of Latin and Greek) is thus apparent.

But there are two other sciences more or less indispensable in this preparation for the concerns of life.—Mathematics and Logic.

Though by mathematics, or the sciences of figure and number, the natural world has been brought under the intellectual and manual power of man, it is not on this account chiefly that its aid is important in intellectual training and discipline. Well hath Plato said—

"Let no man enter the schools of philosophy, who has not previously disciplined his mind by geometry. He considered this science as the first purification of the soul by abstracting the attention from the accidents of the senses." (p. 29.)

The proper province of logic is reasoning.

"Reasoning is the daily and hourly business of our lives, and, whatever our worldly calling—in the pulpit, at the bar, or at the bedside of the patient—we are unceasingly occupied in inferring or proving a something from what is already ascertained or taken for granted. And in the words of an eminent writer on logic, 'To learn to do that well, which every one will and must do, whether well or ill, may surely be considered as an essential part of a liberal education.'" (p. 31.)

To master, therefore, the art of reasoning conclusively, and to guard against those causes which interfere with sound reasoning, "we should especially accustom ourselves to think and reason in precise and steadfast terms."

And here we take the liberty of quoting a rule laid down by Coleridge in his 'Aids to Reflection,' as containing advice by which those who know nothing of logic as a science may avoid the sophisms of others, or of their own minds; an aphorism of constant application in that reasoning which all of us are engaged in silently at the bedside, or in consultations, or in conversations with the sick or with their friends.

"Whether you are reflecting for yourself, or reasoning with another, make it a rule to ask yourself the precise meaning of the word, on which the point in question appears to turn; and if it may be (that is, by writers of authority has been) used in several senses, then ask which of these the word is at present intended to convey."

The professor having thus reached this vantage ground from which can be viewed the requisites of a liberal education, rises to a higher theme, and suspects that already the student who has followed him will discover that among these means of self-culture no place has yet been assigned for truths "that have an especial relation to the moral and spiritual being." Truths which no self-inquirer can fail in discovering to have their "birth, growth and requisite foundation in the whole man, head and heart, and without the possession of which he will live in the eclipses of the better half of his being, moral and intellectual. Not mere truths of the intellect, which in its noblest offspring—Science, is still but a fragment of the whole man. Truths, not left to fortuitous choice, or uncertain proof; but realities, powers of truth, which actuate as well as enlighten, and which are coextensive with our humanity at all times, in all places, and under all circumstances;"

"—Truths that wake
To perish never;
Which neither listlessness, nor mad endeavour,
Nor man, nor boy,
Nor all that is at enmity at joy,
Can utterly abolish or destroy!

Hence, in a season of calm weather,
Though inland far we be,
Our souls have sight of that immortal sea
Which brought us hither,
Can in a moment travel thither—
And see the children sport upon the shore,
And hear the mighty waters rolling evermore." (p. 34.)

And what are these living truths, "which are the light of all our seeing?" Are they not the idea of the good—the idea of the beautiful—the ideas of eternity, immortality, freedom, holiness, happiness, duty,—the idea of our own soul as the reality of which the body is only the covering, "of God as the absolute cause of all reality, Supreme Being, containing all perfection, excluding all want, privation or negation, in the plenitude of goodness, truth and love?" These are the living truths which do actuate man at all times more or less unconsciously, in childhood as high moral instincts by which he is impelled and governed, and which, when reason has become his guide, are to his self-conscious mind, ideas, principles, or final aims, and when sifted by reason he asks for the source of these eternal truths

"that are the life and reality of his spiritual being, where shall he find it but in the Supreme Will causative of all reality, and how name it but the Living Truth,

'—whence the soul
Reason receives; and Reason is her being.'

"But thus conceived there neither is, nor can be, but One Reason;—and, in truth, it is a statement of the Christian doctrine, that the Word by whom all things were made, is essential Light and Life to his creatures;—it is the sublime doctrine revealed by St. John, that the Reason is the light and spiritual presence of the Logos: *τὸ φῶς τὸ ἀληθινὸν, ὃ φωτίζει πάντα ἄνθρωπον ἐρχόμενον εἰς τὸν κόσμον.*" (p. 37.)

Finally, if reason be the sole universal light of man, it follows that it is the essence of all science.

"There are indeed many sciences; still they must all retain the same inherency in, the same known and understood derivation from, the common trunk, of which they are living and growing branches. . . .

"I will conclude by comprising in a few short paragraphs the qualifications of a medical practitioner, considered in relation to the dignity and efficiency of the medical profession according to its ultimate aim.

"I need scarcely indeed dwell upon the possession of technical knowledge and skill; since it is evident that the medical practitioner, who aims at the performance of those duties, which his profession demands, will possess himself of the requisites for its practice, which no honest man would be without. But if I am asked, what are we to understand by the amount of skill and knowledge, which may be justly demanded of the members of a liberal profession, and here of the medical? It is evidently this, that each severally should be capable of applying all the resources of art, which the whole profession can supply. And in this country, and at the present time, in no small proportion of cases, every man, who has fairly and in good earnest availed himself of the advantages, which our medical institutions afford, will have the satisfaction of feeling that he is doing what every other regularly educated physician or surgeon would do under the same or similar circumstances. And where, from the imperfection of our knowledge, we are unable to refer the facts and phenomena to intelligible principles, it should still be our aim and endeavour so to arrange and combine them as to bring them more and more under the conditions, which facilitate the discovery of a principle.

"Hence then, in stating the qualifications of the members of the medical profession, we place as the second requisite scientific insight, or the possession of those laws, or rational grounds, which form at once the principles and ultimate aims of all professional knowledge. Disjoined from the patient and persevering details of observation, the search for facts, and the wakeful attention to them when presented, the healing art would soon fall back to the state, in which during a portion of the middle ages it actually existed, when medicine was little better than a fantastic branch of logic. On the other hand, wholly separated from all speculative science it would necessarily become a mere collection of cases, of facts without any copula that might render them severally or collectively intelligible—nay, without any security that the supposed facts are actually such, or that the most important incidents may not have escaped the notice of the observer. But this is not all. It is not in the nature of the human mind to remain satisfied with the mere record of always imperfect cases. In the absence of insight the imagination takes its place; ostentatious affidavits supply proofs of the efficacy of the medicine and of the *methodus medendi;*

'And puffing quacks confiding dupes allure
'To swear the pill or drop has wrought a cure!'" (pp. 38-40.)

"Lastly;—in order to secure the enlarged education, which is to realize the ardent wish and deliberate scheme of the council of this College for the elevation of the surgical profession,—[a scheme which the reviewer takes the liberty of adding, would have met with the unqualified approbation of the members of that college if it had been carried out in the spirit of a kind and considerate justice to the older surgeons of the country who have grown gray in the actual practice of surgery]—I have only to express my unaltered conviction, strengthened indeed by the success with which the trial has been already attended,—that it is in universities and colleges that a medical education may be best grounded on those universal elements of science, which are the essential constituents of every liberal profession. I hold it indeed scarcely possible that any professional education can be fully accomplished except in such institutions;—where discipline both intellectual and moral, and a pledged direction and supervision of the studies, give the requisite security for its progress and completion;—and where the *Alumni* are induced habitually to regard themselves as members of one body, and to form among themselves a correspondent law of honour, of self-respect, and of respect for each other as fellow-collegians—with the cognate habit of despising the hollow, the tricky, and the ostentatious,—in short, to form that sentiment of honour and gentlemanly feeling, in which the moral life of the individual breathes as in its natural atmosphere, with an unconsciousness, which gives the charm of unaffected manners and conduct." (pp. 38-40.)

We conclude our pleasant task of giving a summary of an Oration so artistic in its conception and execution, so noble in its purpose, so elevated in its thoughts, so correct in its principles, so deeply founded on true science. Where we have not directly quoted we have often conveyed our own reading of the professor's views, often in more common and more intelligible language, in order to accommodate those unaccustomed to its specific phraseology, hoping that this recapitulation may serve as an inducement to many to read the original, which has an Appendix on several metaphysical points, the full understanding of which is necessary to the due comprehension of the lecture.

Such an essay requires careful and even repeated reading, especially by those not conversant with the writings of Coleridge. As an Oration delivered to a body who can hardly be thought to be thus furnished, its value must have consisted in the general impression it conveyed of an earnest religious-minded man, of cultivated intellect, and with the highest spiritual aims, enforcing with eloquent words, and with a chivalrous spirit, his deep convictions that the true way of elevating the medical profession, and making it indeed the calling of a gentleman, was by a comprehensive education that developed harmoniously all the faculties of the man.

If its effect were tested on the mere information each hearer had gained, or in his power of carrying away the line of argument, it might have been pronounced to have failed; but those accustomed to the best examples of parliamentary eloquence, recognised in the orator one whose commanding person, voice, elocution, self-possession, proved him gifted by nature for his office, whilst the general effect of his full-toned and somewhat gorgeous eloquence was one of raised and higher feeling, with a justifiable satisfaction that such a man, so endowed both naturally and by mental culture, should have been found to represent the surgery of England, and to cast over this annual recognition of the genius of Hunter a certain nobility and grandeur, from a gleam of light proceeding from a loftier region of thought and aspiration than that to which the common ordinary man attains.

---

